# Crohn’s disease patients with L4-esophagogastroduodenal phenotype is associated with a better prognosis: A retrospective cohort study

**DOI:** 10.3389/fphar.2022.963892

**Published:** 2022-10-28

**Authors:** Jingrong Weng, Xutao Lin, Xi Chen, Yu-fan Liang, Yu-cheng Xu, Jia-wei Cai, Peng-cheng Lu, Yuming Rong, Yifeng Zou, Lixin Zhu

**Affiliations:** ^1^ Department of Colorectal and Anal Surgery, The Sixth Affiliated Hospital, Sun Yat-sen University, Guangzhou, China; ^2^ Guangdong Institute of Gastroenterology, The Sixth Affiliated Hospital, Sun Yat-sen University, Guangzhou, China; ^3^ Guangdong Provincial Key Laboratory of Colorectal and Pelvic Floor Diseases, The Sixth Affiliated Hospital, Sun Yat-sen University, Guangzhou, China; ^4^ Department of Gastroenterology, The First People's Hospital of Foshan, Foshan, China; ^5^ Department of Very Important Person Region, Sun Yat-sen University Cancer Center, Guangzhou, China

**Keywords:** Crohn’s disease, EGD involvement, Abdominal surgery, Complication, Prognosis

## Abstract

**Background:** In the Montreal classification, L4 Crohn’s disease (CD) is defined as an ileal disease, including L4-esophagogastric duodenum (EGD), L4-jejunum, and L4-proximal ileal involvement. According to the previous studies, the prognosis of L4 disease was worse than that of non-L4 disease. Among L4 diseases, the phenotypes of L4-jejunum and L4-proximal ileum indicated that the risk of abdominal surgery was higher. However, the prognosis of L4-esophagogastroduodenal remains largely elusive. Therefore, we aim to investigate whether the prognosis differs between CD patients with and without esophagogastroduodenal involvement.

**Methods:** In this study, patients with L4-EGD phenotype (*n* = 74) who underwent gastroscopy, ileocolonoscopy, biopsies, and CTE from 2018 to 2020 were compared with L4 non-EGD controls (*n* = 148) who were randomly selected at a ratio of 1:2 in the same period. Demographic information inclusive of disease conduct and location, important points of the surgery, and hospitalization have been collected. The distinction between L4-EGD patients and non-L4-EGD patients was identified by way of multivariable logistic regression analysis. The Kaplan–Meier technique was used to consider the possibility of abdominal surgical operation and complications, observed by means of Cox percentage hazard fashions to decide if L4 EGD independently estimated the endpoints inclusive of the abdominal surgery and the occurrences of complications.

**Results:** L4-EGD group (*n* = 74) had a lower proportion of intestinal fistula than the control group (*n* = 148) (17.6% *versus* 34.5%; *p* = 0.009), and the probabilities of requiring abdominal surgery and multiple abdominal surgeries were also lower (21.6% *versus* 36.5%; *p* = 0.025), (6.8% *versus* 18.9%; *p* = 0.016), respectively. The frequency of hospitalization was lower in the L4-EGD group than in the control group (3-7 *versus* 4–9; *p* = 0.013). L4-EGD phenotype was found to be an independent protective factor for abdominal surgery and intestinal fistula in the Cox regression model, with HRs of 0.536 (95%CI: 0.305–0.940; *p* = 0.030) and 0.478 (95%CI: 0.259–0.881; *p* = 0.018), respectively.

**Conclusion:** Our data suggest that the L4-EGD phenotype may have a better prognosis compared to the Non-L4-EGD phenotype. Our data may advocate a revision of the Montreal classification including separate designations for L4-EGD disease.

## 1 Introduction

Crohn’s disease is a persistent recurrent inflammatory disorder, which mainly affects the gastrointestinal tract with a tendency to development to penetrating and stricturing problems that want a couple of surgical procedures and lifelong drug cure (Chow et al., 2009). CD has long been recognized as a heterogeneous disease with diverse clinical manifestations and features. Parts of many parts of the world are experiencing suffering from CD. (Gasche et al., 2000). In realizing the differences in outcomes between different disease locations, The Vienna classification divided diseases into four categories: L1, terminal ileum (TI) involvement; L2, colonic involvement; L3, ileocolonic disease involving both the TI and T2; and L4, disease proximal to the TI without TI or colonic involvement (Gasche et al., 2000). However, an increasing number of patients with not only L1-3 disease but also L4 disease have been found clinically, which has prompted people to further revise the classification of diseases. So, in 2005, in a new classification of Crohn’s disease, the Montreal classification, L4 disease was redefined as a proximal disease that could coexist with L1-3 diseases (Silverberg et al., 2005). Montreal disorder region classification can now not solely be used to predict the prognosis of Crohn’s disease, however additionally performs a crucial position in scientific trials, affected person counseling, guiding cure, and imparting stratification criteria. Compared to white patients, Chinese patients with Crohn’s disease had a greater percentage (22.7%) of the L4 phenotype, which physically encompasses L4 esophagogastroduodenal (EGD), L4 jejunal, and proximal L4 ileal involvement. (Chow et al., 2009). According to the ACG Clinical Guideline, Endoscopically, Crohn’s disease can be identified by mucosal nodules, ulcers (labial and linear), thickening of the antrum, and duodenal strictures. These histologic alterations can also be seen as granulomatous inflammation, localized occult inflammation of the duodenum, and focal enhancing gastritis after being prepared into a pathologic specimen (Heller et al., 1999; Decker et al., 2001; Van Hogezand et al., 2001; Greuter et al., 2018; Lichtenstein et al., 2018). While frequently the endoscopist would prefer to think of the L4-EGD phenotype when they observe these distinctive results in terms of aphthous ulcers, longitudinal/irregular erosions, ulcers, and bamboo-like look (Dancygier and Frick, 1992; Yokota et al., 1997; Tseng et al., 2007; Sakuraba et al., 2014; Mao et al., 2018).

According to the previous studies, within L4 disease, the phenotype of L4-jejunal and L4-proximal ileal disease indicated a higher risk for abdominal surgery (Lazarev et al., 2013a). However, the prognosis of L4-EGD has been reported with a large variation which remains largely elusive. Similar investigations have not yet revealed whether individuals with L4-EGD phenotypes have different prognoses from those of patients without L4-EGD phenotypes. Exploring the prognostic differences between L4-EGD and non L4-EGD typing is critical since the disease’s phenotype plays a crucial and irreplaceable role in not only predicting prognosis but also directing early aggressive treatment choices for patients. Therefore, this study aimed to discover whether or not there is a difference in outcome between sufferers with CD with an L4-EGD phenotype and sufferers without an L4-EGD phenotype.

## 2 Materials and methods

### 2.1 Population and study design

All patients with the L4-EGD phenotype from all patients with confirmed CD between January 2018 and December 2020 at the Sixth Subsidiary Sun Yat-sen University Hospital, a tertiary referral facility, were included in this observational cohort study that was retrospectively undertaken. L4-EGD-group were compared with controls which were randomly selected at a ratio of 1:2 from this period in the same period. The inclusion criteria were: 1) complete demographic and clinical information with regular clinical follow-up visits; 2) comprehensive examination of the digestive system: patients underwent gastroscopy, ileocolonoscopy, and CTE/MRE (computed tomography enterography and magnetic resonance enterography); 3) underwent biopsies from the esophagus, great curvature of gastric, gastric angle, gastric antrum, duodenal bulb, and descending of duodenum when the gastroscopy was performed.

The following were added as exclusion criteria: 1) the patient’s ultimate diagnosis of unexplained colitis and intestinal illnesses brought on by other potential causes, such as non-steroidal anti-inflammatory drug (NSAID) enteropathy or intestinal tuberculosis; 2) isolated ulcer, localized intestinal wall edema, and no concurrent endoscopically common abnormalities.

The distinction between L4- EGD and non- L4- EGDpatients based on endoscopic, imaging, and histological results were determined in accordance with the definitions given above. At least two independent GI doctors with experience in the treatment of CD evaluated the phenotypes complying with the Montreal description.

### 2.2 Data collection

The information was gathered from our patient CD database. Clinical and demographic data were obtained, including gender [males *versus* female], age at diagnosis [≤ 16 years *versus* >16 years],BMI [≤ 18.5 years *versus* >18.5], nationality, smoke status [smoker *versus* non-smoker], drug treatment policy [steroids, immunomodulators, anti-TNF agents], abdominal complications [intestinal fistula, stenosis or obstruction], perianal complications [perianal fistula, perianal abscess], abdominal surgical history [Yes *versus* > No], perianal surgery history [Yes *versus* > No] and frequency of hospitalization [continuous variable], duration of disease [continuous variable].

### 2.3 Outcome measures and definitions

According to a prior publication, the small intestine was segmented into the terminal ileum, proximal ileum, and jejunum on CTE/MRE imaging (Park et al., 2013). The left lower quadrant was where the proximal ileum was located, while the terminal ileum stretched 10 cm from the ileocecal valve (Ekbom et al., 1991; Levine et al., 2011; Despott and Fraser, 2012; Lazarev et al., 2013a; Park et al., 2013.

To assess the prognosis of patients with EGD involvement, surrogate signs such as intestinal strictures, intestinal and perianal fistulas, perianal abscesses, and the requirement for CD-related abdominal surgery were employed. Any operation that involves any of the following methods was considered abdominal surgery: surgery for fistula or abscess, ileal resection, ileocaecal resection, small bowel resection other than terminal ileum, right or left colectomy, colectomy, proctocolectomy, ileostomy, and colostomy. An upper gastrointestinal endoscopy and biopsy were used to assess the histology of an EGD lesion (Lazarev et al., 2013a).

Those who characterized lesions as: 1) Diagnosed as CD patients. 2) Diagnosed by gastroscopy as gastric ulcer, esophageal ulcer, duodenal ulcer, esophageal stenosis, pyloric stenosis, and duodenal stenosis. It was thought that mucosal erythema was inadequate to prove CD involvement (Lazarev et al., 2013a). 3) Biopsies results showed a large number of acute or chronic lymphocytic plasma cells infiltrated with granuloma formation. 4) CTE/MRE findings included esophagograstroduodel segmental mural thickening or stenosis.

According to the Montreal categorization, disease locations were categorized. In the case of CD, L1 stands for disease in the terminal ileum, L2 for disease in the colon, L3 for ileocolonic disease, and L4 for disease in the jejunum and proximal ileum (Silverberg et al., 2005). Hospitalizations attributable to diseases other than CD were not included.

### 2.4 Statistical analysis

The student’s *t*-test was used to compare two groups’ values for continuous normally distributed variables that were expressed as mean standard deviation. The Mann-Whitney *U* test was used to compare variables with non-normal distribution. As numbers and percentages, discrete data were presented. For categorical variables, chi-squared tests were used. Confounding factors (age, gender, BMI, smoke status, medication) were included in univariate analysis and multivariable analysis. In order to distinguish the two groups, significant predictors of the cumulative chance of abdominal surgery and intestinal fistula were found using multivariable logistic regression models. Their hazard ratios (HRs) and 95% confidence intervals were calculated using Cox proportional hazards models (CIs). SPSS (version 25.0) for Windows was used to conduct statistical analyses above. The cumulative chances of surgery and the cumulative chances of intestinal fistula in various groups were calculated using the Kaplan-Meier curve. The R software (version 4.2.1) was used to conductKaplan-Meier curve. For all analyses, a *p* value of 0.05 was taken as statistical significance.

## 3 Results

### 3.1 Demographics, characteristics, and clinical outcomes between L4-EGD and non-L4-EGD

Baseline demographics, characteristics, and disease features in the experiment group (L4-EGD) and control group (Non-L4-EGD) are summarized in Table1. There are 222 patients included in this study. In total, the median age was 27 [IQR 21-34], with 178 males (80.2%). All 222 patients underwent gastroscopy, ileocolonoscopy, biopsies, and CTE. Overall, 74 patients developed with L4-EGD phenotype, and 96, 95, 91, and 88 were diagnosed with L1, L2, L3, L4-jejunal, and L4-proximal ileal phenotype, respectively. Among them, 124, 44, 54,150 were classified as nonstricturing nonpenetrating (B1), stricturing (B2), penetrating (B3) and perianal disease, respectively. In total, 64 of 222 patients (28.8%) developed intestinal fistula while 94 of 222 patients (42.3%) developed stenosis or obstruction and 134 of 222 (60.4%) patients developed a perianal fistula and 70 of 222 patients (31.5%) developed a perianal abscess. 70 of 222 patients (31.5%) underwent abdominal surgery which included small bowel resection and colorectal resection while 33 of 222 patients (14.9%) underwent multiple abdominal surgeries. In addition 99 of 222 (44.6%) underwent fistula or abscess surgery. The median frequency of hospitalization was 6 [IQR 4-9]. (Table1).

**TABLE 1:** Patient demographic and disease characteristics of CD patients with EGD involvement *versus* control CD patients without EGD involvement.

Clinical characteristics	Total (*N* = 222)	Non-EGD involvement (*N* = 148)	EGD involvement (*N* = 74)	*p* value
Gender				0.812
Male	178/222 (80.2%)	118/148 (79.7%)	60/74 (81.1%)	
Female	44/222 (19.8%)	30/148 (20.3%)	14/74 (18.9%)	
Age at the time of diagnosis, yrs [median, IQR, range]	27, 21–34	27, 21–36	27, 22–33	0.904
Age at the time of diagnosis, yrs, *n* (%)				1.000
16 or less	15/222 (6.8%)	10/148 (6.8%)	5/74 (6.8%)	
More than 16	207/222 (93.2%)	138/148 (93.2%)	69/74 (93.2%)	
BMI [kg/m2 ], n (%)				0.849
18.5 or less	121/222 (54.5%)	80/148 (54.1%)	41/74 (55.4%)	
More than 18.5	101/222 (45.5%)	68/148 (45.9%)	33/74 (44.6%)	
Smoking status at latest follow-up				1.000
Non-smoker	204/222 (91.9%)	136/148 (91.9%)	68/74 (91.9%)	
Smoker	18/222 (8.1%)	12/148 (8.1%)	6/74 (8.1%)	
Montreal classification of disease location, n (%)				
L1 (terminal ileal)	96/222 (43.2%)	62/148 (41.9%)	34/74 (46.0%)	0.565
L2 (colonic)	35/222 (15.8%)	25/148 (16.9%)	10/74 (13.5%)	0.515
L3 (ileocolonic)	91/222 (41.0%)	61/148 (41.2%)	30/74 (40.5%)	0.923
L4 (L4-jejunal and L4-proximal ileal)	88/222 (39.6%)	60/148 (40.5%)	28/74 (37.8%)	0.698
Montreal classification of disease behavior, n (%)				
B1 (nonstricturing, nonpenetrating)	124/222 (55.9%)	70/148 (59.7%)	50/74 (67.6%)	0.013
B2 (Stricturing)	44/222 (19.8%)	31/148 (20.9%)	13/74 (17.6%)	0.552
B3 (penetrating)	54/222 (24.3%)	43/148 (29.1%)	11/74 (14.9%)	0.020
P (perianal disease)	150/222 (67.6%)	94/148 (64.5%)	54/74 (73.0%)	0.224
Complication, n (%)				
Intestinal Fistula	64/222 (28.8%)	51/148 (34.5%)	13/74 (17.6%)	0.009
Stenosis/Obstruction	94/222 (42.3%)	63/148 (42.6%)	31/74 (41.9%)	0.923
Perianal fistula	134/222 (60.4%)	84/148 (56.8%)	50/74 (67.6%)	0.121
Perianal abscess	70/222 (31.5%)	47/148 (31.8%)	23/74 (33.1%)	0.919
Medication, *n* (%)				
Steroids	74/222 (33.3%)	52/148 (35.1%)	22/74 (29.7%)	0.421
Immunomodulators	145/222 (65.3%)	102/148 (68.9%)	43/74 (58.1%)	0.111
Anti-TNF agents	105/222 (47.3%)	66/148 (44.6%)	39/74 (52.7%)	0.254
Abdominal surgery, *n* (%)				0.025
NO	152/222 (68.5%)	94/148 (63.5%)	58/74 (78.4%)	
YES	70/222 (31.5%)	54/148 (36.5%)	16/74 (21.6%)	
Multiple abdominal surgeries, *n* (%)				0.016
1 or 0	189/222 (85.1%)	120/148 (81.8%)	69/74 (93.2%)	
more than 1	33/222 (14.9%)	28/148 (18.9%)	5/74 (6.8%)	
Fistula/abscess surgery, *n* (%)				0.252
NO	123/222 (55.4%)	86/148 (58.1%)	37/74 (50.0%)	
YES	99/222 (44.6%)	62/148 (41.9%)	37/74 (50.0%)	
Hospitalizations, [median, IQR, range]	6, 4–9	6, 4–9	5, 3–7	0.013

Data are mean ± standard deviation, median (interquartile range, IQR), or n/N (%). *p* values comparing patients with EGD, involvement and patients without EGD, involvement are from Student *t* test, Man-Whitney *U* test, Chi-Square test or Fisher’s exact test, as appropriate. BMI = Body Mass Index. The differing denominators used indicate missing data.

### 3.2 Comparison between patients with and without EGD involvement

**FIGURE 1:**
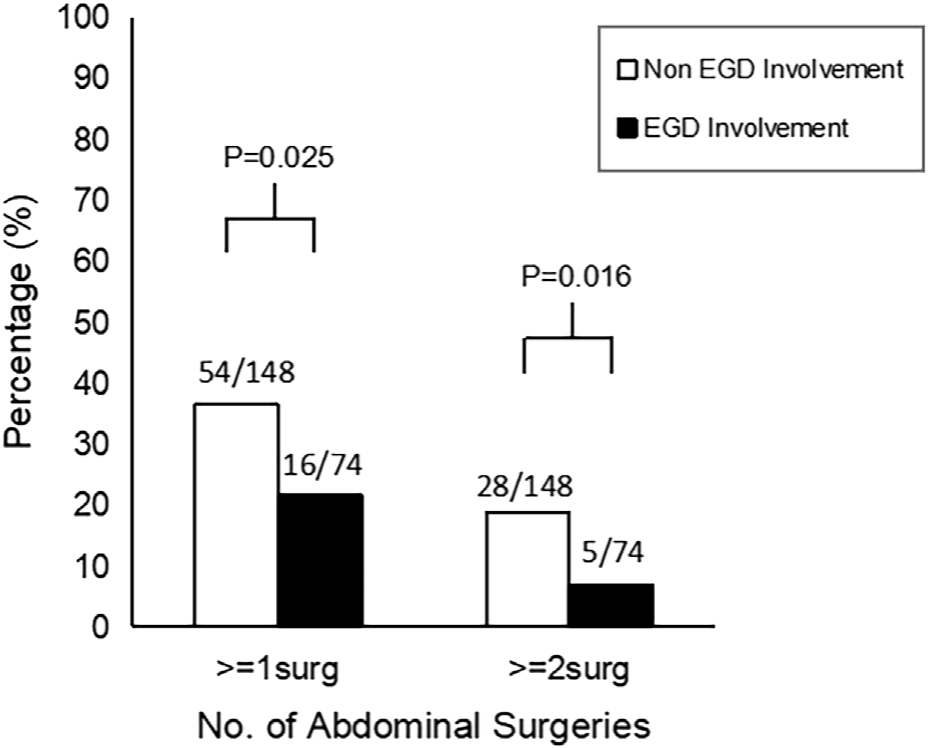
Abdominal surgery rates in EGD involvement and Non-EGD involvement.

L4-EGD contrasted with non-L4-EGD. The demographics, characteristics, and clinical outcomes of patients with and without L4-EGD involvement are shown in Table 1. There were no significant differences in age (*p* = 0.904), gender (*p* = 0.904), BMI (*p* = 0.849), smoke status (*p* = 1.000), steroids treatment (*p* = 0.421), immunomodulators treatment (*p* = 0.111), anti-TNF agents treatment (*p* = 0.254), stenosis or obstruction (*p* = 0.923), perianal surgery (*p* = 0.252), no difference was observed in the comparison of disease locations. Surprisingly, although there were no significant differences in the comparison of disease locations, more B3 phenotypes were found in the Non-L4-EGD group than in the L4-EGD group (29.1% *versus* 14.9%; *p* = 0.020). In contrast, B1 phenotypes were more common in the L4-EGD group than in the Non-L4-EGD group (67.6% *versus* 59.7%; *p* = 0.013). When compared to patients in the Non-L4-EGD group, patients in the L4-EGD group had a lower percentage of intestinal fistulas (17.6 percent *versus* 34.5 percent; *p* = 0.009). There were significantly lower proportions of patients who underwent abdominal surgery in the L4-EGD group than in the Non-L4-EGD group (21.6% *versus* 36.5%; *p* = 0.025). Moreover, the L4-EGD group had a lower proportion of patients who underwent multiple abdominal surgeries (6.8% *versus* 18.9%; *p* = 0.016) (Figure 1). There was a significantly lower frequency of hospitalization in the L4-EGD group than in the Non-L4-EGD group (3-7 *versus* 4–9; *p* = 0.013). A similar result was found in the stratified analysis of L4 that the proportion of B3 phenotypes and the proportion of intestinal fistula in the Non-L4-EGD group were higher than that in the L4-EGD group (31.7% *versus* 14.9%; *p* = 0.020) and (38.3% *versus* 17.6%; *p* = 0.007), respectively (Table2).

**TABLE 2:** Stratified analysis of the clinical outcomes of L4-jejunal and L4-proximal ileal without EGD Involvement VS. EGD involvement in all the patients.

Clinical characteristics	Total (*N* = 134)	Non-EGD involvement (*N* = 60)	EGD involvement (*N* = 74)	*p* Value
Montreal classification of disease behavior, n (%)				
B1 (nonstricturing, nonpenetrating)	80/134 (59.7%)	30/60 (50.0%)	50/74 (67.6%)	0.039
B2 (Stricturing)	24/134 (17.9%)	11/60 (18.3%)	13/74 (17.6%)	0.908
B3 (penetrating)	30/134 (22.4%)	19/60 (31.7%)	11/74 (14.9%)	**0.020**
P (perianal disease)	90/134 (67.2%)	36/60 (60.0%)	54/74 (73.0%)	0.112
Complication, *n* (%)				
Intestinal Fistula	36/134 (26.9%)	23/60 (38.3%)	13/74 (17.6%)	**0.007**
Stenosis/Obstruction	56/134 (41.8%)	27/60 (45.0%)	31/74 (41.9%)	0.718
Perianal fistula	82/134 (61.2%)	32/60 (53.3%)	50/74 (67.6%)	0.093
Perianal abscess	41/134 (30.6%)	18/60 (30.0%)	23/74 (33.1%)	0.893
Abdominal surgery, n (%)	35/134 (26.1%)	21/60 (35.0%)	16/74 (21.6%)	0.085
Multiple abdominal surgeries, n (%)	14/134 (10.4%)	9/60 (15.0%)	5/74 (6.8%)	0.121

Data are mean ± standard deviation, median (interquartile range, IQR), or n/N (%). p values comparing patients with EGD involvement and patients without EGD involvement are from Student *t* test, Man-Whitney *U* test, Chi-Square test or Fisher’s exact test, as appropriate. The differing denominators used indicate missing data. P values are significant.

### 3.3 Independent predictors of abdominal surgery and intestinal fistula

Univariate and multivariate logistic regression analysis for the main outcome of abdominal surgery was carried out, as could be seen in Supplementary Table S1, after taking into account all factors that could be connected to abdominal surgery. We found that abdominal surgery was significantly associated with BMI and L4-EGD phenotype in univariate logistic regression analysis. After adjusting for confounding factors in multivariate logistic regression analysis, BMI ≥18.5 and L4-EGD phenotype were still significantly with abdominal surgery with an adjusted ORs of 0.523 (95%CI: 0.285–0.958; *p* = 0.036) and 0.466 (95%CI: 0.242–0.898; *p* = 0.023), respectively.

We then performed a univariate logistic regression analysis to identify the risk factors and protective factors for disease behavior and intestinal fistula. After including all variables that were possibly associated with intestinal fistula, we found that intestinal fistula was significantly associated with L4-EGD phenotype with ORs of 0.405 (95%CI: 0.204–0.807; *p* = 0.010). After adjusting the influence of confounding factors in multivariate logistic regression, the L4-EGD phenotype was still significantly associated with intestinal fistula with ORs of 0.396 (95%CI: 0.197–0.798; *p* = 0.007), showing that L4-EGD phenotype might be a protective factor for abdominal surgery and intestinal fistula (Supplementary Table 2).

### 3.4 Impact of esopahgograstroduodenal involvement on abdominal surgery and abdominal complications

**FIGURE 2:**
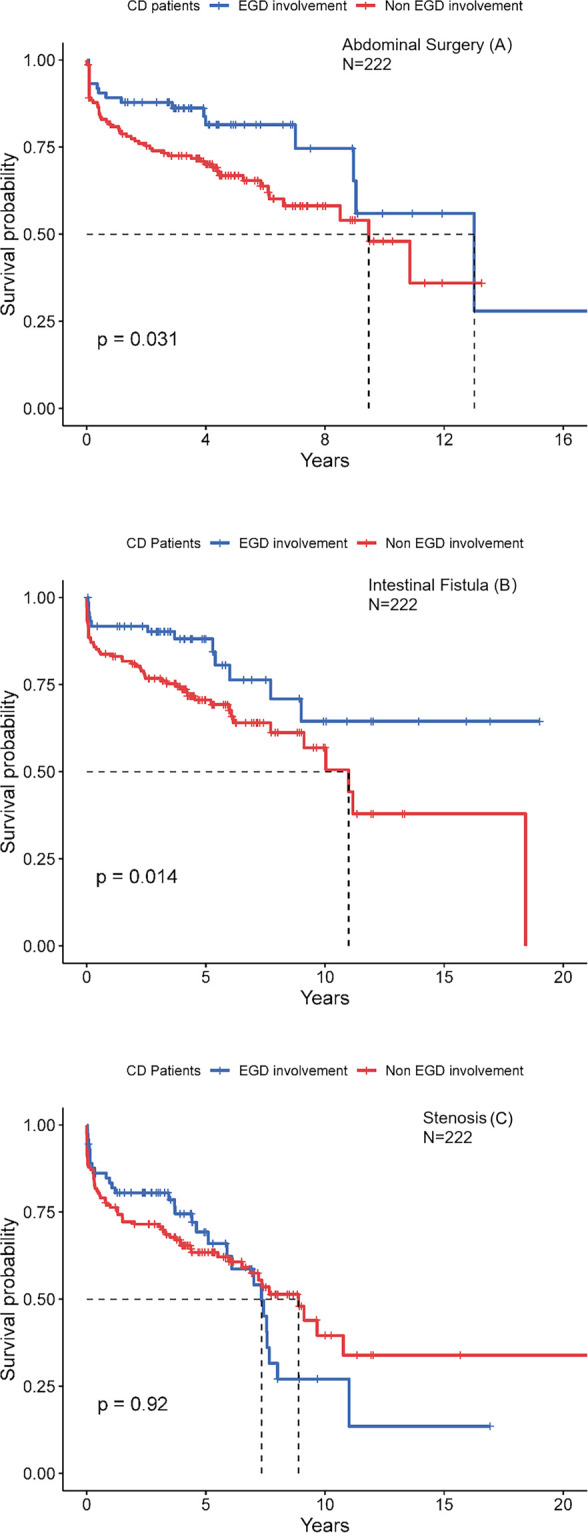
Kaplan-Meier analysis for the occurrence of abdominal surgery **(A)**, internal fistula **(B)**, stenosis **(C)**.

To investigate the impact of the L4-EGD phenotype on disease course, we analyzed the clinical course of all L4-EGD patients at the time of CD diagnosis compared with controls without the L4-EGD phenotype. In Figure 2, Kaplan-Meier curves for abdominal surgery-free survival and complication-free survival are shown. An improvement in outcome was seen in the L4-EGD group according to the Kaplan-Meier analysis for abdominal surgery-free survival [log-rank test *p* = 0.031]. Additionally, the Kaplan-Meier analysis for intestinal fistula-free survival indicated a tendency towards significance with a superior result in the L4-EGD group [log-rank test *p* = 0.008]. However, Kaplan-Meier analysis for stenosis-free survival showed no differences between the two groups [log-rank test *p* = 0.200].

Hazard ratios for the development of complications and abdominal surgery are summarized in Table 3 and Table 4. Again, the L4-EGD phenotype was independently predictive of abdominal surgery and intestinal fistula with an adjusted HRs of 0.536 (95%CI: 0.305–0.940; *p* = 0.030) and 0.478 (95%CI: 0.259–0.881; *p* = 0.018), respectively, showing that L4-EGD phenotype was found to be an independent protective factor to predict the progression to abdominal surgery and intestinal fistula.

**TABLE 3:** Analysis of possible risk factors predicting abdominal surgery using the Cox Model (*N* = 222).

	Multivariable HR (95%CI)	*p* value
Age at the time of diagnosis	0.911 (0.325–2.553)	0.860
Gender	0.690 (0.398–1.196)	0.186
BMI	0.599 (0.363–0.988)	**0.045**
EGD involvement	0.536 (0.305–0.940)	**0.030**
Smoking status	—	—
Steroids	—	—
Immunomodulators	—	—
Anti-TNF agents	—	—
L1 (terminal ileal)	—	—
L2 (colonic)	—	—
L3 (ileocolonic)	—	—
L4 (L4-jejunal and L4-proximal ileal)	0.889 (0.543–1.453)	0.638

HR, hazard ratio; CI, confidence interva, Cox Model were adjusted for age, gender, BMI, EGD involvement, smoking status, medication and disease location. P values are significant.

**TABLE 4:** Analysis of possible risk factors predicting intestinal fistula using the Cox Model (*N* = 222).

	Multivariable HR (95%CI)	*p* Value
Age at the time of diagnosis	0.608 (0.146–2.543)	0.496
Gender	0.586 (0.336–1.024)	0.061
BMI	0.657 (0.392–1.102)	0.112
EGD involvement	0.478 (0.259–0.881)	**0.018**
Smoking status	—	—
Steroids	—	—
Immunomodulators	—	—
Anti-TNF agents	—	—
L1 (terminal ileal)	—	—
L2 (colonic)	—	—
L3 (ileocolonic)	—	—
L4 (L4-jejunal and L4-proximal ileal)	1.141 (0.691–1.884)	0.112

HR, hazard ratio; CI, confidence interva, Cox Model were adjusted for age, gender, BMI, EGD involvement, smoking status, medication and disease location. P values are significant.

## 4 Discussion

The ileocecal area is the most prevalent location for CD lesions, while they can develop elsewhere throughout the GI system. Chinese CD patients have a higher proportion of L4 phenotypes (22.7%) than do white patients (Papp et al., 2008; Chow et al., 2009; Lazarev et al., 2013b). Some present study results have shown that patients with the L4 phenotype might more likely to undergo multiple abdominal surgeries and develop abdominal complications such as intestinal fistula and stenosis or obstruction (Chow et al., 2009; Lazarev et al., 2013a). Nearly 70% of the L4 group’s major surgeries over the course of 5 years were major operations (Chow et al., 2009). However, A study from Johns Hopkins University recently reported that only patients with jejunal involvement (L4 jejunum) showed this connection, whereas those with L4-EGD involvement showed the opposite association. (Atreya and Siegmund, 2021). Additionally, individuals with L4 jejunalopathy underwent several abdominal operations more frequently than those with non-L4 ileopathy, in addition to stricturing behavior (Atreya and Siegmund, 2021). Chen and colleagues also reported a similar conclusion that patients with the L4 subtypes L4-EGD, L4-jejunum, and L4-proximal ileum might have significant variations in their clinical prognoses (Lazarev et al., 2013a). Although currently grouped all upper GI involvement by the Montreal classification system as L4 disease, the prognosis of L4-EGD, L4-jejunal, and L4-proximal ileum disease are very different from one another. There have been no relevant large-sample clinical studies on patients with L4-EGD phenotypes. Thus, we carried out this study.

In our study, patients with L4-EGD phenotype (*n* = 74) who underwent gastroscopy, ileocolonoscopy, biopsies, and CT enteroclysis in the Sixth affiliated hospital of Sun Yat-sen University from 2018 to 2020 were compared with controls (*n* = 148) in the same period. Eventually, 222 patients were included in this study. We found that there was no significant difference between L4-EGD patients and controls in gender, age, BMI, disease location, and medical management including using steroids, immunomodulators, and anti-TNF agents. L4-EGD group had a lower proportion of intestinal fistula than the control group, and the probability of requiring abdominal surgery and multiple abdominal surgeries was also lower. What’s more, the frequency of hospitalization was lower in the L4-EGD group than in the control group. In addition, a higher proportion of B1 (nonstricturing, nonpenetrating) and a lower proportion of B3 (penetrating) were found in the L4-EGD group, the same as the proportion of complications. Multivariate logistic regression analysis found that the L4-EGD phenotype was a protective factor for abdominal surgery and intestinal fistula. L4-EGD phenotype was also found to be an independent protective factor to predict the progression of abdominal surgery and intestinal fistula in the Cox regression model.

To the best of our knowledge, our study is the first to show that individuals with the L4-EGD phenotype had a higher likelihood of having a good prognosis, which is different from other studies. Different from patients with L4-jejunum phenotype and patients with L4-proximal ileum phenotype, patients with L4-EGD phenotype have a lower chance of developing complications and have a lower rate of requiring multiple abdominal surgeries. Our study indicated that the upper gastrointestinal tract Phenotype of CD has been associated with early surgery and further hospitalization due to the L4-jejunal phenotype and L4-proximal ileum phenotype but the frequency of early surgery and additional hospitalization is lower among the L4-EGD phenotypes. We previously believed that L4 disease would be predisposed to more severe symptoms because L4 disease has a wider involvement so CD patients with the L4 phenotype are potential targets for the top-down strategy. However, our study showed that this medical management should not be used in patients with L4-EGD phenotype.

Our conclusions are crucial for two reasons. First off, it could affect the planning and analysis of the future investigation of the relationship between esophagogastroduodenal participation in disease site correlations with genotypes, serology, and other biomarkers. Indeed, a recent German study outlines the differences on various levels between ileal and colonic disease by comparing the differences in physiological evidence, gut microbiota, intestinal mucus layer, epithelial cells, T cells and cytokine profiles, leukocyte trafficking, clinical implications, the clinical course of the disease, treatment of disease, which confirmed that CD patients with ileal involvement and CD patients with colonic involvement are two different phenotypes (Atreya and Siegmund, 2021). Thus, CD patients with esophagogastroduodenal involvement may be proved as a different phenotype by future studies. Second, the L4 phenotype is a prospective target for the top-down method due to their elevated risk of acquiring complicationsand have a high rate of requiring multiple abdominal surgeries. However, this drug therapy is not entirely suitable for CD patients with L4-EGD phenotype, because CD patients with L4-EGD phenotype have a better prognosis than CD patients without L4-EGD phenotype. It can reduce the overtreatment of CD patients with L4-EGD phenotype.

Our study had several limitations. First, despite the relatively large number of patients included, our study is a single-center retrospective study, and the conclusion of our study needs to be verified by a large sample of prospective clinical studies. RCT studies can adjust confounding factors such as the influence of different kinds of races. Second, our study is aretrospective cohort study but not a population-based study, although there were no significant differences in age, gender, BMI, smoke status, or even disease location. Nonetheless, prospective studies investigating the demographics and disease location difference between the L4-EGD group and the non-L4-EGD group are needed. Third, Our study only compared the differences between the L4-EGD group and the non-L4-EGD group in the prognosis and clinical course of the disease, so this conclusion needs to be determined by physiological evidence, gut microbiota, intestinal mucus layer, epithelial cells, T cells, and cytokine profiles, leukocyte trafficking in the future studies. What’s more, there may be a confounding factor that contributes to the decreased surgical rates in patients with the L4-EGD phenotype: surgeons are hesitant to operate on patients with Treitz proximal disease since patients with EGD involvement may be responsive to medicinal treatment readily. However, the fact that the L4-EGD group had a lower proportion of intestinal fistula than the control group and the fact that the L4-EGD group also had a lower frequency of hospitalization in contrast to the Non-L4-EGD strongly argue in favor of the advice that L4-EGD phenotype is indeed associated with better prognosis, regardless of surgical considerations.

## 5 Conclusion

In conclusion, the L4-EGD phenotype may have a better prognosis compared to the Non-L4-EGD phenotype. Patients with L4-EGD phenotype are associated with a lower risk for intestinal fistula, abdominal surgery, and further hospitalizations. Moreover, Patients with the L4-EGD phenotype have a lower proportion of B3 (penetrating) phenotype and have a higher proportion of B1 (nonstricturing, nonpenetrating) than patients without the L4-EGD phenotype. Therefore, based on the findings of the current study, we might propose further modifying the Montreal categorization to separate the L4 phenotype into two distinct categories, including L4-EGD and non-L4-EGD, if the findings of additional investigations are consistent. Although our study discovered that this occurrence differs from Montreal typing, additional cellular and molecular testing is still required to properly corroborate this finding.

## Data Availability

The original contributions presented in the study are included in the article/Supplementary Material, further inquiries can be directed to the corresponding authors.
