# Supplementary material for: Crohn’s disease patients with L4-esophagogastroduodenal phenotype is associated with a better prognosis: A retrospective cohort study
Source: Front Pharmacol. 2022 Oct 28;13:963892. doi: 10.3389/fphar.2022.963892 (PMC9649486; doi:10.3389/fphar.2022.963892)
Supplement: Supplementary file 1 [file Table1.DOCX]

| Supplemental Table 1, Univariate and multivariate logistic regression model for prediction of abdominal surgery between patients with EGD involvement and control CD patients without EGD involvement (N=222) | | | | |
| --- | --- | --- | --- | --- |
|  | Univariable OR  (95%CI) | p  value | Multivariable OR (95%CI) | P  value |
| **Age at the time of diagnosis** | 0.777 (0.238-2.531) | 0.675 | 0.627 (0.185-2.129) | 0.699 |
| 16 or less |  |  |  |  |
| More than 16 |  |  |  |  |
| **Gender** | 0.673 (0.339-1.338) | 0.259 | 0.699 (0.346-1.414) | 0.319 |
| Male |  |  |  |  |
| Female |  |  |  |  |
| **BMI [kg/m2 ]** | 0.556 (0.310-0.995) | **0.048** | 0.523 (0.285-0.958) | **0.036** |
| 18.5 or less |  |  |  |  |
| More than 18.5 |  |  |  |  |
| **EGD involvement** | 0.480 (0.251-0.917) | **0.026** | 0.466 (0.242-0.898) | **0.023** |
| NO |  |  |  |  |
| YES |  |  |  |  |
| **Smoking status** | 0.597 (0.189-1.885) | 0.380 | / | / |
| Non-smoker |  |  |  |  |
| Smoker |  |  |  |  |
| **Medication** |  |  |  |  |
| Steroids | 0.881 (0.481-1.615) | 0.683 | / | / |
| Immunomodulators | 1.237 (0.677-2.262) | 0.489 | / | / |
| Anti-TNF agents | 1.639 (0.927-2.900) | 0.089 | / | / |
| **Montreal classification of disease location** |  |  |  |  |
| L1 | 1.064 (0.601-1.883) | 0.832 | / | / |
| L2 | 0.994 (0.457-2.164) | 0.989 | / | / |
| L3 | 0.942 (0.529-1.677) | 0.839 | / | / |
| L4-jejunal, L4-proximal ileal | 0.937 (0.524-1.674) | 0.825 | 0.816 (0.444-1.499) | 0.513 |
| OR=odds ratio. CI=confidence interval. confidence interva, Multivariable logistic regression were adjusted for age, gender, BMI, EGD involvement, smoking status, medication and disease location. | | | | |

| Supplemental Table 2, Univariate and multivariate logistic regression model for prediction of intestinal fistula between patients with EGD involvement and control CD patients without EGD involvement (N=222) | | | | |
| --- | --- | --- | --- | --- |
|  | Univariable OR (95%CI) | p value | Multivariable OR (95%CI) | p value |
| **Age at the time of diagnosis** | 0.360 (0.079-1.642) | 0.187 | 0.317 (0.066-1.514) | 0.150 |
| 16 or less |  |  |  |  |
| More than 16 |  |  |  |  |
| **Gender** | 0.570 (0.285-1.139) | 0.111 | 0.561 (0.274-1.148) | 0.114 |
| Male |  |  |  |  |
| Female |  |  |  |  |
| **BMI [kg/m2 ]** | 0.631 (0.348-1.144) | 0.129 | 0.614 (0.330-1.143) | 0.124 |
| 18.5 or less |  |  |  |  |
| More than 18.5 |  |  |  |  |
| **EGD involvement** | 0.405 (0.204-0.807) | **0.010** | 0.396 (0.197-0.798) | **0.010** |
| NO |  |  |  |  |
| YES |  |  |  |  |
| **Smoking status** | 0.945 (0.323-2.769) | 0.918 | / | / |
| Non-smoker |  |  |  |  |
| Smoker |  |  |  |  |
| **Medication** |  |  |  |  |
| Steroids | 0.968 (0.522-1.794) | 0.917 | / | / |
| Immunomodulators | 1.124 (0.608-2.080) | 0.709 | / | / |
| Anti-TNF agents | 1.164 (0.651-2.082) | 0.608 | / | / |
| **Montreal classification of disease location** |  |  |  |  |
| L1 | 0.595 (0.325-1.087) | 0.091 | / | / |
| L2 | 0.985 (0.443-2.190) | 0.971 | / | / |
| L3 | 1.678 (0.933-3.017) | 0.084 | / | / |
| L4-jejunal, L4-proximal ileal | 1.390 (0.772-2.504) | 0.272 | 1.232 (0.665-2.281) | 0.508 |
|  |  |  |  |  |
| OR=odds ratio. CI=confidence interval. confidence interva, Multivariable logistic regression were adjusted for age, gender, BMI, EGD involvement, smoking status, medication and disease location. | | | | |
|  |  |  |  |  |
|  |  |  |  |  |
|  |  |  |  |  |


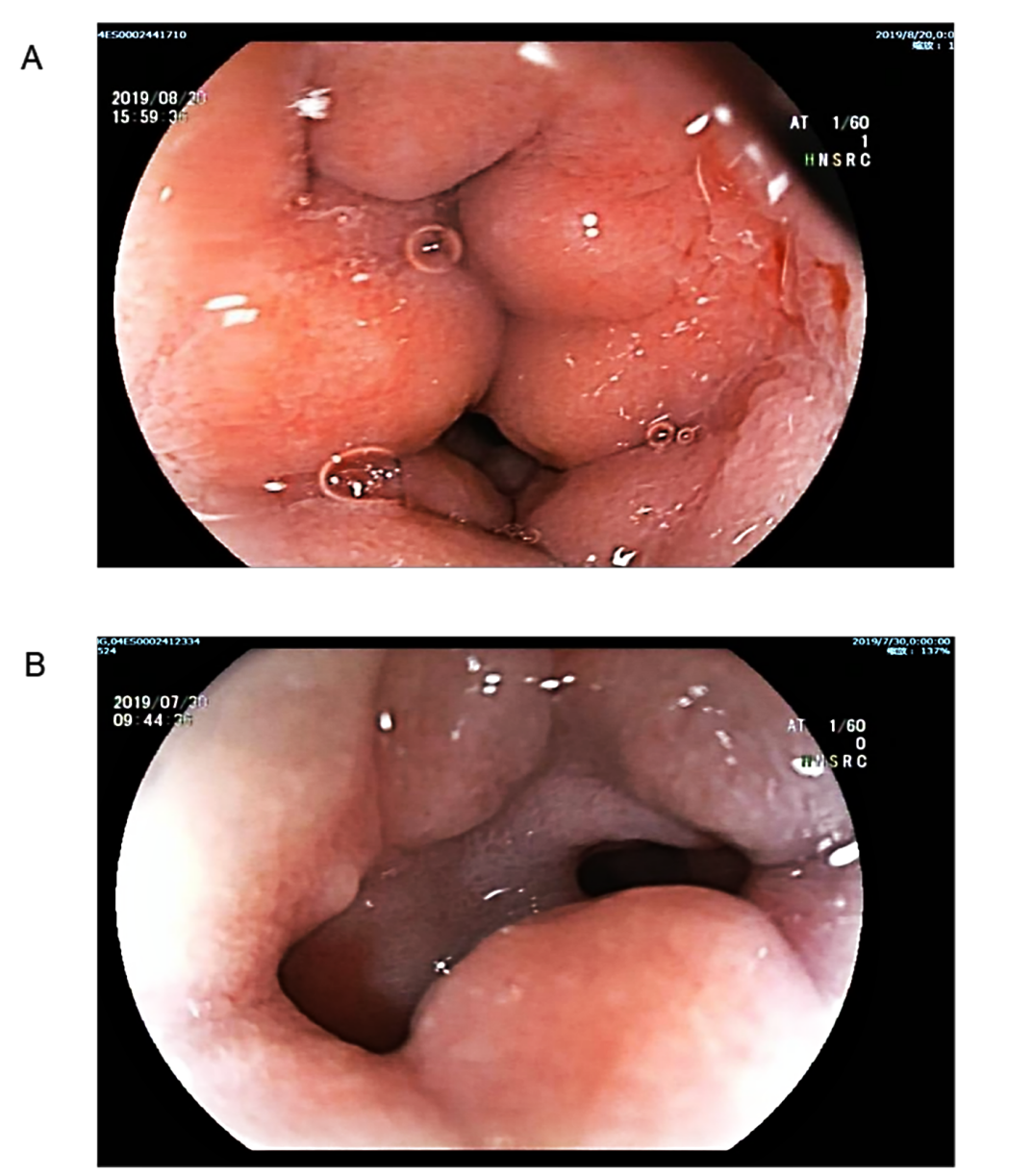


Supplemental Figure 1, Endoscopic features of duodenal strictures of CD
